# Atrial fibrillation status and functional capacity in transthyretin cardiac amyloid cardiomyopathy

**DOI:** 10.1016/j.ijcha.2026.102037

**Published:** 2026-11-04

**Authors:** Antonio Kösters, Nikita Ermolaev, Mahshid Eslami, Lisa Seilinger, Nima Rassoulpour, Mariusz Grzeda, Roza Badr Eslam, Christoph Schukro

**Affiliations:** a Division of Cardiology, Department of Internal Medicine II, Medical University of Vienna, 1090 Vienna, Austria; b Department of Cardiac and Thoraco-Aortic Surgery, Medical University of Vienna, 1090 Vienna, Austria; c Department of Clinical Pharmacology, Medical University of Vienna, 1090 Vienna, Austria; d University of Bristol, Department of Population Health Sciences, Bristol, United Kingdom

**Keywords:** Transthyretin cardiac amyloidosis, Atrial fibrillation, Functional capacity, Six-minute walk test, Cardiopulmonary exercise testing, Exercise intolerance

## Abstract

**Background:**

Atrial fibrillation is common in transthyretin cardiac amyloid cardiomyopathy (ATTR-CM), but its association with objective physical performance across atrial fibrillation status groups remains unclear. We investigated the association between atrial fibrillation status and functional capacity in ATTR-CM, using six-minute walk distance (6MWD) as the primary endpoint.

**Methods:**

Consecutive adults with confirmed ATTR-CM evaluated between June 2018 and October 2025 were included and categorized as no atrial fibrillation, paroxysmal atrial fibrillation, or permanent atrial fibrillation. The primary endpoint was six-minute walk distance for submaximal functional capacity assessment. Secondary exploratory endpoints included cardiopulmonary exercise testing (CPET) parameters and NT-proBNP. Multivariable linear regression adjusted for age, sex, body mass index, and NYHA functional class; sensitivity analyses additionally considered beta-blocker use, rhythm-device status, ATTR subtype, left ventricular ejection fraction, and log-transformed NT-proBNP.

**Results:**

Among 156 patients, 82 had no atrial fibrillation, 24 had paroxysmal atrial fibrillation, and 50 had permanent atrial fibrillation. Six-minute walk distance was available in 136 patients and differed across atrial fibrillation status groups: 432.9 ± 122.4 m, 309.2 ± 111.0 m, and 378.8 ± 108.7 m, respectively. After adjustment, AF status remained associated with 6MWD (overall *p* = 0.031), driven by lower adjusted values in paroxysmal versus no AF. Significant CPET-derived differences were present in unadjusted analyses but attenuated after adjustment for clinical parameters.

**Conclusions:**

In ATTR-CM, atrial fibrillation status is associated with functional capacity, with six-minute walk distance providing the most robust parameter. Submaximal functional assessment may capture clinically relevant rhythm-related physical impairment in ATTR-CM.

## 1 Introduction

Cardiac amyloidosis is an infiltrative cardiomyopathy caused by extracellular deposition of misfolded amyloid fibrils within the myocardium. The two clinically dominant forms are immunoglobulin light-chain amyloidosis and transthyretin amyloid cardiomyopathy (ATTR-CM), with the latter comprising wild-type and hereditary forms. In ATTR-CM, progressive myocardial infiltration leads to increased ventricular wall thickness, diastolic dysfunction, impaired ventricular compliance, elevated filling pressures and, ultimately, heart failure with reduced exercise tolerance [1], [2]. Once considered a rare disease, ATTR-CM is increasingly recognized as an important cause of heart failure, particularly among older patients with preserved or mildly reduced ejection fraction. Improved awareness, non-invasive bone scintigraphy, cardiac magnetic resonance imaging and disease-modifying therapies have increased the clinical relevance of identifying functional impairment and disease progression in this population [3], [4], [5], [6], [7], [8].

Atrial fibrillation is one of the most frequent rhythm disorders in cardiac amyloidosis and is particularly common in ATTR-CM. Amyloid infiltration, increased atrial wall stiffness, elevated filling pressures and progressive atrial remodelling may all contribute to the development of atrial arrhythmias [9], [10]. Previous studies have reported a high prevalence of atrial fibrillation in ATTR-CM, and atrial fibrillation has been associated with more advanced disease, increased thromboembolic risk and worse clinical outcomes [11], [12], [13], [14]. However, atrial fibrillation is not a uniform entity. Patients with paroxysmal and those with persistent or permanent atrial fibrillation may differ with regard to rhythm stability, rate control, atrial mechanical function, chronotropic response and treatment exposure. Despite this, the relationship between atrial fibrillation status and objective measures of physical performance in ATTR-CM remains incompletely characterized [15].

Functional capacity is a central clinical feature in ATTR-CM. Exercise intolerance is often one of the earliest and most disabling manifestations of the disease and reflects the combined impact of impaired cardiac output reserve, chronotropic limitation, pulmonary vascular involvement, skeletal muscle dysfunction, comorbidities and frailty [16]. Objective evaluation of physical performance is therefore important both for clinical phenotyping and for the assessment of disease severity. Cardiopulmonary exercise testing (CPET) provides a comprehensive physiological assessment of exercise capacity, including peak oxygen uptake, ventilatory efficiency and chronotropic response, and has increasingly been used to characterize exercise limitation in cardiac amyloidosis [17], [18], [19], [20]. Recent work has shown that patients with ATTR-CM demonstrate markedly reduced peak VO₂ and abnormal ventilatory responses, supporting the role of CPET as a detailed tool for physiological assessment [21], [22].

In contrast, the six-minute walk test (6MWT) is a simple, widely available measure of submaximal functional capacity [23]. Although less mechanistic than CPET, it may better reflect integrated daily-life performance in older and multimorbid heart failure populations. In ATTR-CM, six-minute walk distance (6MWD) has gained importance as a clinically meaningful endpoint, including in therapeutic trials and observational studies [6], [7], [8], [24]. Recent data have demonstrated that 6MWD is prognostically relevant in ATTR-CM and may help identify patients at increased risk of adverse outcomes [24], [25]. Moreover, in heart failure populations, the 6MWT and CPET provide complementary rather than interchangeable information, as walking performance may capture functional limitations not fully reflected by maximal exercise testing [26].

Whether atrial fibrillation status (i.e. paroxysmal, permanent or no atrial fibrillation) is associated with impaired physical performance in ATTR-CM, and whether this association is better captured by 6MWT or CPET-derived parameters, remains unclear. This question is clinically relevant because atrial fibrillation is highly prevalent in ATTR-CM, potentially modifiable in selected patients, and may contribute to functional impairment beyond conventional echocardiographic markers such as left ventricular ejection fraction or left atrial size. Therefore, the present study aimed to investigate the association between atrial fibrillation status and physical performance in patients with ATTR-CM, using 6MWD as the primary endpoint, as well as CPET-derived parameters and NT-proBNP as secondary exploratory measures.

## 2 Methods

This prospective observational cohort study included 156 consecutive adult patients with confirmed transthyretin cardiac amyloid cardiomyopathy (ATTR-CM) evaluated between June 2018 and October 2025. Patients were enrolled in institutional cardiomyopathy and cardiopulmonary exercise testing registries and contributed to a shared analysis dataset. Eligible patients were adults aged ≥18 years with confirmed ATTR-CM and available baseline atrial fibrillation status. For the primary analysis, patients were required to have available baseline 6MWD.

ATTR-CM was diagnosed according to contemporary clinical practice, integrating clinical assessment, imaging, biomarker evaluation and, where appropriate, histology and/or radionuclide scintigraphy. Diagnostic classification into wild-type ATTR-CM and variant ATTR-CM was based on genetic testing and standard clinical work-up. Patients with light-chain amyloidosis were excluded from this analysis. Patients were also excluded if they were unable to perform functional testing or had clinical conditions expected to limit exercise performance independently of cardiac disease, including severe pulmonary disease and advanced orthopaedic or neurological limitations.

### 2.1 Ethical approval

The study was conducted in accordance with the Declaration of Helsinki. Ethical approval was obtained from the Ethics Committee of the Medical University of Vienna (EK 1918/2019). All participants provided written informed consent prior to registry inclusion.

### 2.2 Atrial fibrillation status classification

Patients were categorized according to baseline atrial fibrillation status into three groups: no atrial fibrillation, paroxysmal and permanent atrial fibrillation. For this study, patients documented as having persistent atrial fibrillation were assigned to the permanent atrial fibrillation group when the treating team had accepted sustained AF as the ongoing rhythm strategy and no further rhythm-control attempts were planned. This operational, study-specific classification was based on the clinical management decision and did not equate persistent and permanent AF on the basis of arrhythmia duration alone. Atrial fibrillation status was determined from medical records, electrocardiograms, Holter monitoring reports and documented rhythm history. Paroxysmal atrial fibrillation was defined as documented intermittent atrial fibrillation with restoration of sinus rhythm, whereas permanent atrial fibrillation was defined as sustained atrial fibrillation accepted as the ongoing rhythm strategy. Patients without documented atrial fibrillation were classified as having no atrial fibrillation.

### 2.3 Six-minute walk test

The primary endpoint was 6MWD, analyzed as a continuous variable. The 6MWT was performed as part of routine baseline functional assessment according to standardized local procedures. Patients were instructed to walk as far as possible within six minutes, and the total distance covered was recorded in meters. 6MWD was selected as the primary endpoint because it reflects submaximal functional capacity and represents a clinically intuitive measure of everyday physical performance in patients with heart failure and ATTR-CM. Only measurements recorded as part of the baseline clinical assessment were used; follow-up measurements were not substituted for missing baseline values.

### 2.4 Cardiopulmonary exercise testing

Symptom-limited cardiopulmonary exercise testing was performed on upright cycle ergometers using individualized ramp protocols targeting an exercise duration of approximately 8–12 min. Breath-by-breath gas exchange analysis was conducted using calibrated metabolic carts according to standard procedures. Peak oxygen uptake was defined as the highest 30-s averaged VO₂ value obtained during peak exercise and was expressed in absolute terms, relative to body weight, and as percentage of predicted peak VO₂ using established reference equations.

Peak workload was recorded in watts and expressed as absolute and percentage predicted values. Respiratory exchange ratio, peak heart rate and percentage of predicted maximal heart rate were recorded. Predicted maximal heart rate was calculated using the Fox formula, defined as 220 minus age. Ventilatory efficiency was expressed as the VE/VCO₂ slope and was derived from the linear relationship between minute ventilation and carbon dioxide production from rest to the respiratory compensation point. Haemodynamic monitoring was limited to standard non-invasive measurements, including electrocardiography and blood pressure assessment at rest, during exercise and recovery.

CPET-derived parameters were analyzed as secondary exploratory endpoints to provide physiological characterization of exercise limitation across atrial fibrillation status groups.

### 2.5 Echocardiography, biomarkers and clinical variables

Baseline echocardiographic parameters included left ventricular ejection fraction, interventricular septal thickness, systolic pulmonary artery pressure, global longitudinal strain and left atrial diameter where available. Laboratory variables included N-terminal pro-B-type natriuretic peptide, estimated glomerular filtration rate and haemoglobin. NT-proBNP was log-transformed for regression analyses because of its skewed distribution.

Clinical variables included age, sex, body mass index, New York Heart Association functional class, ATTR type, relevant comorbidities, beta-blocker use, anticoagulation, loop diuretic therapy and cardiac rhythm-device status (i.e. pacemaker or implantable cardioverter-defibrillators, with or without cardiac resynchronization therapy).

### 2.6 Statistical analysis

Continuous variables are presented as mean ± standard deviation or median with interquartile range, depending on distribution. Categorical variables are presented as counts and percentages. Baseline characteristics were compared across atrial fibrillation status groups using analysis of variance or Kruskal–Wallis tests for continuous variables, and chi-square or Fisher's exact tests for categorical variables, as appropriate.

The primary analysis assessed the association between atrial fibrillation status and 6MWD using linear regression. Atrial fibrillation status was modelled as a categorical variable with no atrial fibrillation as the reference category. The main multivariable model adjusted for age, sex, body mass index and New York Heart Association functional class. The overall association between atrial fibrillation status and 6MWD was assessed using the omnibus *p*-value for the atrial fibrillation status term. Adjusted mean 6MWD values by atrial fibrillation status were estimated from the main multivariable model.

Several sensitivity analyses were performed to evaluate the robustness of the primary finding. First, beta-blocker use was added to the main model. Second, the analysis was repeated after excluding patients receiving beta-blockers. Third, rhythm-device status was added to the main model, and the analysis was repeated after excluding patients with rhythm devices. Fourth, ATTR type was added to the main model, and an additional analysis was restricted to patients with wild-type ATTR-CM. Finally, an exploratory disease-severity model additionally included left ventricular ejection fraction and log-transformed NT-proBNP. This model was interpreted cautiously because of the reduced number of complete cases.

Secondary exploratory analyses evaluated the association between atrial fibrillation status and CPET-derived parameters, including peak VO₂, percentage of predicted peak VO₂, peak workload, percentage of predicted peak workload and VE/VCO₂ slope. These analyses were performed using linear regression models analogous to the primary analysis. Because CPET parameters were secondary exploratory endpoints and were expected to be influenced by disease severity, these results were interpreted as hypothesis-generating.

Analyses used complete cases for the outcome and covariates included in each model; missing values were not imputed. The main adjusted 6MWD model included 133 patients because three of the 136 patients with baseline 6MWD lacked NYHA class. Characteristics of patients with and without baseline 6MWD were summarized descriptively (Supplementary Table S2).

A two-sided *p*-value <0.05 was considered statistically significant. No formal adjustment for multiple testing was applied because the primary endpoint was prespecified and secondary analyses were exploratory. Descriptive statistics and univariable group comparisons were performed using IBM SPSS Statistics. Multivariable regression analyses and figure generation were performed using Python with standard statistical packages.

## 3 Results

A total of 156 patients with confirmed transthyretin cardiac amyloid cardiomyopathy were included in the present analysis. Of these, 82 patients had no documented atrial fibrillation, 24 had paroxysmal atrial fibrillation and 50 had permanent atrial fibrillation, corresponding to 52.6%, 15.4% and 32.1% of the cohort, respectively. Six-minute walk distance was available in 136 patients, including 71 patients without atrial fibrillation, 21 patients with paroxysmal atrial fibrillation and 44 patients with permanent atrial fibrillation.

Baseline 6MWD was unavailable in 20 of 156 patients (12.8%). Retrospective review of the clinical records identified follow-up 6MWT measurements in 17 of these patients, obtained approximately 6 or 12 months after treatment initiation. These measurements were not substituted for baseline values because they reflected functional status during follow-up after treatment exposure. The remaining three patients died before a follow-up 6MWT was obtained. The reasons why baseline walking assessments were absent could not be established retrospectively. All 20 patients without baseline 6MWD had CPET data, including peak VO₂, peak workload, respiratory exchange ratio and VE/VCO₂ slope.

A descriptive comparison of patients with and without baseline 6MWD is provided in Supplementary Table S2. Among patients with recorded NYHA class, NYHA class III was more frequent in those without baseline 6MWD (7/15, 46.7%) than in those with baseline 6MWD (23/133, 17.3%). NYHA class itself was also more often unavailable in patients without baseline 6MWD (5/20, 25.0% vs 3/136, 2.2%). Mean peak VO₂ was 14.4 ± 4.6 versus 15.4 ± 4.5 mL/kg/min, respectively. These descriptive differences do not establish the mechanism of missingness or exclude selection bias.

Baseline characteristics differed across atrial fibrillation status groups (Table 1). Patients with paroxysmal and permanent atrial fibrillation were significantly older than patients without atrial fibrillation. Body mass index did not differ significantly across groups. Most patients were male in all three groups, with no significant difference in sex distribution. Wild-type ATTR-CM represented the dominant ATTR subtype across the cohort.

**Table 1:** Baseline characteristics according to atrial fibrillation status.

Variable	Overall	No AF	Paroxysmal AF	Permanent AF	*P* value
Cohort size and demographics					
N	156	82	24	50	
Age, years	77.4 ± 10.2	74.4 ± 12.5	80.2 ± 4.5	80.9 ± 5.4	<0.001
Body mass index, kg/m^2^	25.5 ± 3.7	25.9 ± 4.1	25.5 ± 2.8	24.8 ± 3.5	0.244
Male sex	139/156 (89.1%)	71/82 (86.6%)	21/24 (87.5%)	47/50 (94.0%)	0.400
ATTR-CM type and functional status					
Wild-type ATTR-CM	143/156 (91.7%)	72/82 (87.8%)	24/24 (100.0%)	47/50 (94.0%)	0.126
NYHA class I	40/148 (27.0%)	29/78 (37.2%)	3/24 (12.5%)	8/46 (17.4%)	0.015
NYHA class II	78/148 (52.7%)	38/78 (48.7%)	12/24 (50.0%)	28/46 (60.9%)	
NYHA class III	30/148 (20.3%)	11/78 (14.1%)	9/24 (37.5%)	10/46 (21.7%)	
Comorbidities					
Hypertension	110/155 (71.0%)	55/81 (67.9%)	17/24 (70.8%)	38/50 (76.0%)	0.611
Coronary heart disease	53/155 (34.2%)	29/81 (35.8%)	9/24 (37.5%)	15/50 (30.0%)	0.741
Stroke	10/154 (6.5%)	4/81 (4.9%)	3/24 (12.5%)	3/49 (6.1%)	0.415
Diabetes mellitus	27/155 (17.4%)	20/81 (24.7%)	1/24 (4.2%)	6/50 (12.0%)	0.031
COPD	14/155 (9.0%)	4/81 (4.9%)	3/24 (12.5%)	7/50 (14.0%)	0.173
Medication and rhythm-device status					
Anticoagulation	88/154 (57.1%)	22/80 (27.5%)	22/24 (91.7%)	44/50 (88.0%)	<0.001
Beta-blocker use	79/155 (51.0%)	32/81 (39.5%)	14/24 (58.3%)	33/50 (66.0%)	0.010
Rhythm drug use	13/154 (8.4%)	3/80 (3.8%)	3/24 (12.5%)	7/50 (14.0%)	0.091
Loop diuretic use	81/154 (52.6%)	33/80 (41.2%)	16/24 (66.7%)	32/50 (64.0%)	0.013
Rhythm device	23/153 (15.0%)	5/80 (6.2%)	6/24 (25.0%)	12/49 (24.5%)	0.006
Laboratory parameters					
Haemoglobin, g/dL	13.9 ± 1.6	13.9 ± 1.6	13.8 ± 1.5	13.9 ± 1.7	0.986
eGFR, mL/min/1.73 m^2^	79.2 ± 13.5	83.2 ± 12.6	74.5 ± 15.1	75.4 ± 12.7	0.006
NT-proBNP, ng/L	1835 [866, 3222]	1283 [608, 2464]	2404 [1703, 5718]	2806 [1697, 3839]	<0.001
Echocardiographic parameters					
Interventricular septum, mm	19.8 ± 4.5	19.5 ± 4.8	21.2 ± 4.3	19.6 ± 4.2	0.385
LVEF, %	51.4 ± 11.6	55.0 ± 10.7	45.5 ± 12.7	49.4 ± 11.0	0.012
sPAP, mmHg	47.4 ± 15.1	47.2 ± 17.6	50.3 ± 15.8	46.4 ± 12.4	0.737
LV GLS, %	−11.5 ± 6.9	−12.7 ± 7.7	−8.6 ± 8.5	−11.2 ± 4.1	0.193
Left atrial diameter, mm	61.3 ± 9.4	60.5 ± 7.3	65.3 ± 6.1	60.5 ± 12.6	0.225

Values are mean ± SD, median [IQR], or n/N (%), as appropriate. *P* values refer to comparisons across the three AF status groups using ANOVA or Kruskal-Wallis tests for continuous variables and chi-square or Fisher's exact tests for categorical variables, as appropriate. AF, atrial fibrillation; ATTR-CM, transthyretin cardiac amyloid cardiomyopathy; eGFR, estimated glomerular filtration rate; GLS, global longitudinal strain; LVEF, left ventricular ejection fraction; NT-proBNP, N-terminal pro-B-type natriuretic peptide; sPAP, systolic pulmonary artery pressure. ANOVA, analysis of variance; COPD, chronic obstructive pulmonary disease; IQR, interquartile range; NYHA, New York Heart Association; SD, standard deviation.

Functional status also differed according to atrial fibrillation status. NYHA functional class distribution was significantly different across groups (*p* = 0.015), with a higher proportion of NYHA class III patients among those with paroxysmal atrial fibrillation. As expected, oral anticoagulation use was more frequent in patients with atrial fibrillation, and beta-blocker use, loop diuretic therapy and cardiac rhythm-device status also differed across groups. As only six patients with paroxysmal atrial fibrillation had such a device implanted, we had no relevant information regarding arrhythmia burden.

Regarding laboratory parameters, estimated glomerular filtration rate was lower and NT-proBNP was significantly different in patients with atrial fibrillation compared with those without atrial fibrillation (Table 1). Left ventricular ejection fraction was significantly lower in patients with atrial fibrillation, whereas left atrial diameter did not differ significantly across groups.

### 3.1 6MWD across atrial fibrillation status groups

6MWD differed significantly across atrial fibrillation status groups in the unadjusted analysis. Patients without atrial fibrillation had the highest walking distance, with a mean 6MWD of 432.9 ± 122.4 m. Patients with paroxysmal atrial fibrillation had the lowest walking distance, with a mean of 309.2 ± 111.0 m, whereas patients with permanent atrial fibrillation showed intermediate values, with a mean of 378.8 ± 108.7 m (overall *p* < 0.001).

In unadjusted linear regression, atrial fibrillation status was significantly associated with 6MWD (overall *p* < 0.001). Compared with patients without atrial fibrillation, paroxysmal atrial fibrillation was associated with a 123.7 m lower 6MWD, while permanent atrial fibrillation was associated with a 54.0 m lower 6MWD. After adjustment for age and sex, the overall association between atrial fibrillation status and 6MWD remained significant (overall *p* = 0.001).

Results of 6MWD by atrial fibrillation status are summarized in Table 2. In the prespecified main multivariable model adjusted for age, sex, body mass index and NYHA functional class, atrial fibrillation status remained independently associated with 6MWD (overall *p* = 0.031). Adjusted mean six-minute walk distances were 411.2 m for no atrial fibrillation, 344.3 m for paroxysmal atrial fibrillation and 401.5 m for permanent atrial fibrillation (Fig. 1). The overall association was mainly driven by lower adjusted 6MWD in the paroxysmal atrial fibrillation group. Detailed regression estimates are shown in Table 3.

**Table 2:** Six-minute walk distance by atrial fibrillation status.

Measure	No AF	Paroxysmal AF	Permanent AF	Overall *P* value
N with 6MWT	71	21	44	
Observed 6MWD, m, mean ± SD	432.9 ± 122.4	309.2 ± 111.0	378.8 ± 108.7	<0.001
Adjusted mean 6MWD, m (95% CI)	411.2 (387.4 to 435.1)	344.3 (301.1 to 387.5)	401.5 (370.9 to 432.0)	0.031

Adjusted means were estimated from the main multivariable model adjusted for age, sex, body mass index and NYHA functional class. 6MWD, six-minute walk distance; 6MWT, six-minute walk test; AF, atrial fibrillation; CI, confidence interval. BMI, body mass index; NYHA, New York Heart Association; SD, standard deviation.

**Fig. 1:**
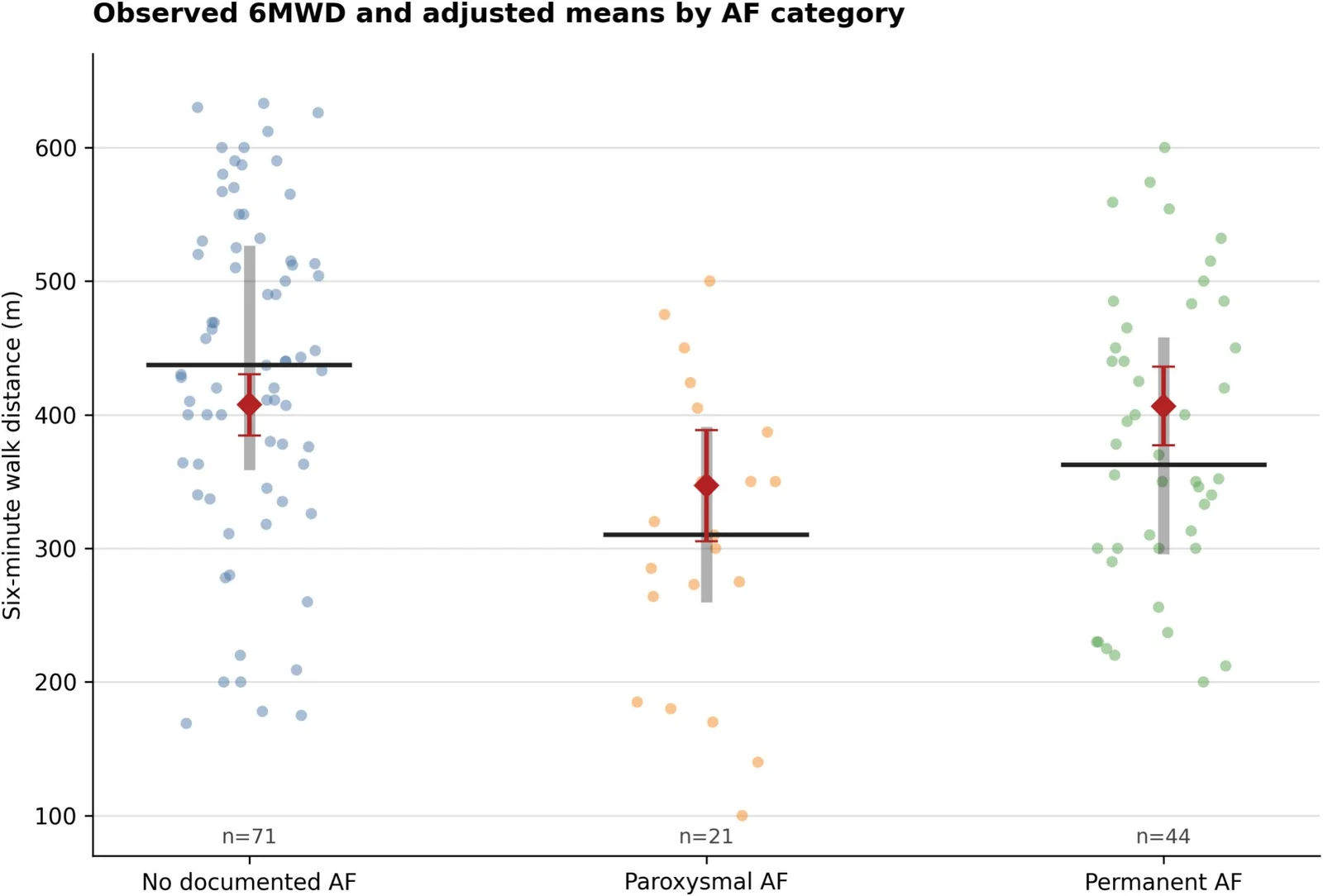
Observed 6MWD and adjusted means by AF category. Dots show individual observed 6MWD values; black bars show medians and interquartile ranges. Red diamonds and error bars show adjusted means and 95% confidence intervals from the primary model (*n* = 133), adjusted for age, sex, body mass index, and ordinal NYHA class. AF, atrial fibrillation; 6MWD, six-minute walk distance. NYHA, New York Heart Association.

**Table 3:** Linear regression models for the association between atrial fibrillation status and six-minute walk distance.

Model	N	Overall AF status P	Paroxysmal AF vs no AF, B (95% CI)	P value	Permanent AF vs no AF, B (95% CI)	P value	R^2^
Unadjusted	136	<0.001	−123.7 (−180.9 to −66.5)	<0.001	−54.0 (−98.2 to −9.8)	0.017	0.129
Model 1: age + sex	136	0.001	−104.6 (−160.6 to −48.7)	<0.001	−35.3 (−79.4 to 8.8)	0.116	0.209
Main Model 2: age + sex + BMI + NYHA	133	0.031	−66.9 (−117.2 to −16.6)	0.010	−9.7 (−49.4 to 29.9)	0.628	0.400
Exploratory severity model: + LVEF + log NT-proBNP	59	0.007	−111.3 (−182.9 to −39.7)	0.003	−1.9 (−57.5 to 53.8)	0.947	0.625

Regression coefficients represent the adjusted mean difference in 6MWD in metres compared with the no AF group. The overall AF status P value is the omnibus P value for the categorical AF status term. CI, confidence interval; NT-proBNP, N-terminal pro-B-type natriuretic peptide. AF, atrial fibrillation; B, unstandardized regression coefficient; BMI, body mass index; LVEF, left ventricular ejection fraction; NYHA, New York Heart Association; R^2^, coefficient of determination; 6MWD, six-minute walk distance.

R^2^ values reflect model fit within the complete-case sample available for each model and should not be compared directly across models because sample sizes differ.

Sensitivity analyses supported the robustness of the association between atrial fibrillation status and 6MWD (Table 4). After additional adjustment for beta-blocker use, atrial fibrillation status remained borderline associated with six-minute walk distance (overall *p* = 0.050), and paroxysmal atrial fibrillation remained associated with lower six-minute walk distance. In an analysis excluding patients receiving beta-blockers, the association between atrial fibrillation status and 6MWD was strengthened (overall *p* = 0.004).

**Table 4:** Sensitivity analyses for the primary six-minute walk distance endpoint.

Sensitivity model	N	Overall AF status P	Paroxysmal AF vs no AF, B (95% CI)	P value	Permanent AF vs no AF, B (95% CI)	P value
Main model	133	0.031	−66.9 (−117.2 to −16.6)	0.010	−9.7 (−49.4 to 29.9)	0.628
+ beta-blocker use	133	0.050	−55.6 (−104.2 to −7.0)	0.025	2.8 (−35.9 to 41.5)	0.888
Excluding beta-blocker users	68	0.004	−126.8 (−203.8 to −49.8)	0.002	7.9 (−50.7 to 66.6)	0.788
+ rhythm device status	132	0.055	−59.9 (−110.9 to −8.8)	0.022	−3.0 (−43.6 to 37.6)	0.884
Excluding rhythm-device patients	113	0.186	−54.5 (−113.9 to 4.9)	0.072	−3.9 (−49.0 to 41.2)	0.864
+ ATTR type	133	0.034	−66.5 (−117.2 to −15.9)	0.010	−9.8 (−49.6 to 30.0)	0.628
Wild-type ATTR-CM only	124	0.037	−64.9 (−116.1 to −13.7)	0.013	−5.3 (−46.5 to 36.0)	0.801
+ LVEF + log NT-proBNP	59	0.007	−111.3 (−182.9 to −39.7)	0.003	−1.9 (−57.5 to 53.8)	0.947

All sensitivity models are based on the main model adjusted for age, sex, body mass index and NYHA functional class unless otherwise specified. Regression coefficients represent adjusted mean differences in 6MWD in metres compared with the no AF group. The model including LVEF and log NT-proBNP was interpreted as exploratory due to reduced complete-case sample size. AF, atrial fibrillation; ATTR-CM, transthyretin cardiac amyloid cardiomyopathy; B, unstandardized regression coefficient; BMI, body mass index; CI, confidence interval; LVEF, left ventricular ejection fraction; NT-proBNP, N-terminal pro-B-type natriuretic peptide; NYHA, New York Heart Association; 6MWD, six-minute walk distance.

Additional adjustment for rhythm-device status yielded a similar direction of effect, with an overall *p*-value of 0.055. When patients with rhythm devices were excluded, the association was attenuated and no longer statistically significant (overall *p* = 0.186), although the estimated difference for paroxysmal atrial fibrillation remained directionally consistent. Adjustment for ATTR subtype did not substantially change the result (overall *p* = 0.034), and the association remained significant when the analysis was restricted to patients with wild-type ATTR-CM (overall *p* = 0.037).

In an exploratory disease-severity model additionally including left ventricular ejection fraction and log-transformed NT-proBNP, atrial fibrillation status remained significantly associated with 6MWD (overall *p* = 0.007). However, this model included only 59 patients with complete data and was therefore interpreted as exploratory. Across sensitivity analyses, the estimated association between paroxysmal atrial fibrillation and lower six-minute walk distance remained directionally consistent, whereas permanent atrial fibrillation was not independently associated with lower 6MWD after adjustment.

### 3.2 CPET and secondary exploratory endpoints

CPET parameters differed across atrial fibrillation status groups in unadjusted analyses. Peak VO₂ was highest in patients without atrial fibrillation and lower in both atrial fibrillation groups. Mean peak VO₂ (*p* = 0.002) and percentage of predicted peak VO₂ (*p* = 0.011) were significantly higher in patients without atrial fibrillation than in patients with paroxysmal or permanent atrial fibrillation.

Peak workload also differed significantly across groups. Patients without atrial fibrillation achieved a higher peak workload than patients with paroxysmal or permanent atrial fibrillation (*p* < 0.001). Percentage of predicted peak workload was similarly lower in both atrial fibrillation groups compared with patients without atrial fibrillation (*p* = 0.020). Ventilatory efficiency, expressed as the VE/VCO₂ slope, also differed across atrial fibrillation status groups, with higher values in patients with atrial fibrillation (*p* = 0.009). Peak respiratory exchange ratio and peak heart rate did not differ significantly across groups.

In exploratory multivariable models adjusted for age, sex, body mass index and NYHA functional class, most CPET-derived associations were attenuated and were no longer statistically significant. The strongest residual CPET signal was observed for peak workload, where the overall atrial fibrillation status association was borderline (overall *p* = 0.072), with lower standardized peak workload in permanent atrial fibrillation compared with no atrial fibrillation. Peak VO₂, percentage of predicted peak VO₂, percentage of predicted peak workload and VE/VCO₂ slope were not independently associated with atrial fibrillation status in the clinically adjusted exploratory models.

Log-transformed NT-proBNP remained significantly associated with atrial fibrillation status in exploratory adjusted analyses (overall *p* = 0.007). This association was primarily driven by higher log-transformed NT-proBNP in patients with permanent atrial fibrillation compared with those without atrial fibrillation (Table 5).

**Table 5:** Secondary exploratory functional and biomarker endpoints.

Outcome	No AF	Paroxysmal AF	Permanent AF	Unadjusted P	Adjusted overall AF P
Peak VO₂, mL/kg/min	16.5 ± 4.8	13.8 ± 3.5	14.0 ± 3.9	0.002	0.154
Peak VO₂, % predicted	76.9 ± 19.2	68.5 ± 15.4	68.0 ± 16.7	0.011	0.409
Peak workload, W	90.9 ± 32.5	69.6 ± 24.5	69.7 ± 29.9	<0.001	0.072
Peak workload, % predicted	88.9 ± 29.9	77.1 ± 22.0	75.8 ± 26.6	0.020	0.572
VE/VCO₂ slope	36.5 ± 8.0	40.3 ± 6.8	40.7 ± 8.8	0.009	0.179
NT-proBNP, ng/L, median [IQR]	1283 [608, 2464]	2404 [1703, 5718]	2806 [1697, 3839]	<0.001	0.007

Values are mean ± SD except for NT-proBNP, which is presented as median [IQR]. Unadjusted *P* values refer to comparisons across AF status groups. Adjusted overall AF *P* values were obtained from exploratory models adjusted for age, sex, body mass index and NYHA functional class. NT-proBNP was log-transformed for adjusted regression analyses. CPET, cardiopulmonary exercise testing; VE/VCO₂, ventilatory equivalent for carbon dioxide; VO₂, oxygen uptake. Complete-case sample sizes were *n* = 148 for peak VO₂ and peak workload, *n* = 146 for VE/VCO₂ slope, and *n* = 98 for NT-proBNP. AF, atrial fibrillation; BMI, body mass index; IQR, interquartile range; NT-proBNP, N-terminal pro-B-type natriuretic peptide; NYHA, New York Heart Association; SD, standard deviation.

## 4 Discussion

In this prospective cohort of patients with ATTR-CM, atrial fibrillation status was associated with functional capacity as assessed by 6MWD. Patients without atrial fibrillation had the highest, and those with paroxysmal atrial fibrillation had the lowest 6MWD. The association between atrial fibrillation status and six-minute walk distance remained significant after adjustment for age, sex, body mass index and NYHA functional class. This finding was directionally consistent across several sensitivity analyses including adjustment for beta-blocker use, rhythm-device status and ATTR subtype, and in an analysis restricted to patients with wild-type ATTR-CM. In contrast, CPET-derived differences were observed in unadjusted and basic adjusted analyses but were largely attenuated after broader adjustment for clinical parameters. These findings suggest that atrial fibrillation status is associated with a clinically relevant functional phenotype in ATTR-CM, with the most robust parameter captured by submaximal walking performance rather than maximal exercise testing.

Atrial fibrillation is highly prevalent in ATTR-CM and has been linked to disease stage, atrial remodelling, thromboembolic risk and adverse clinical outcomes [11]. The high frequency of atrial fibrillation in ATTR-CM likely reflects the combined effects of amyloid infiltration, elevated filling pressures, atrial mechanical dysfunction and progressive atrial structural remodelling. In this context, atrial fibrillation may impair functional capacity through several mechanisms, including loss of atrial contribution to ventricular filling, irregular ventricular response, chronotropic limitation, impaired cardiac output reserve and treatment-related effects such as rate-limiting medication. Our findings extend previous work by focusing specifically on physical performance across atrial fibrillation status groups rather than on atrial fibrillation as a binary comorbidity [15].

The observation that the strongest reduction in 6MWD was seen in patients with paroxysmal atrial fibrillation is noteworthy and should be interpreted cautiously. While permanent atrial fibrillation might intuitively be expected to be associated with the greatest functional impairment, paroxysmal atrial fibrillation may represent a clinically unstable rhythm phenotype characterized by intermittent loss of atrial mechanical function, variable rate control and fluctuating symptoms. In contrast, patients with permanent atrial fibrillation may be more consistently rate-controlled and may be physically better adapted to chronic atrial fibrillation. Limited subgroup size may also contribute to this pattern. Therefore, the finding should not be interpreted as evidence that paroxysmal atrial fibrillation is intrinsically more harmful than permanent atrial fibrillation in ATTR-CM, but rather as a signal that atrial fibrillation status may capture clinically relevant heterogeneity in functional performance.

The 6MWT has gained increasing relevance in ATTR-CM because it reflects integrated submaximal performance and has been associated with prognosis and disease progression [24], [25]. Unlike CPET, which provides detailed physiological assessment under maximal or near-maximal exercise conditions, the 6MWT captures a more pragmatic measure of daily-life functional capacity. This may be particularly relevant in ATTR-CM, a disease that commonly affects older patients with multiple comorbidities, frailty, chronotropic abnormalities and extracardiac limitations. The persistence of the 6MWD association after adjustment for clinical parameters suggests that submaximal functional assessment may be especially sensitive to the clinical burden associated with atrial fibrillation status.

In contrast, CPET-derived parameters were less independently robust in multivariable analyses. This does not mean that CPET is not informative in ATTR-CM. Nevertheless, CPET is a valuable tool for characterizing the mechanisms of exercise limitation, including reduced peak oxygen uptake, impaired workload, ventilatory inefficiency and abnormal chronotropic response. Previous studies have shown that CPET parameters are impaired in cardiac amyloidosis and may carry prognostic information [16], [17], [21]. However, CPET variables are closely related to global disease severity, age, NYHA class, cardiac output reserve, pulmonary pressures, rhythm status, medication use and patient effort. Therefore, attenuation after adjustment is not unexpected and may reflect the complex overlap between atrial fibrillation, ATTR-CM severity and maximal exercise physiology. In the present study, CPET should therefore be interpreted as exploratory physiological characterization, whereas 6MWD represents the primary clinically robust functional endpoint.

The relationship between atrial fibrillation status and functional capacity was not fully explained by conventional echocardiographic markers. Although left ventricular ejection fraction differed across atrial fibrillation groups in baseline comparisons, the association between atrial fibrillation status and six-minute walk distance remained present in an exploratory model additionally including left ventricular ejection fraction and log-transformed NT-proBNP. Left atrial diameter also did not appear to fully account for the observed functional differences. This should be interpreted with caution, as echocardiographic and biomarker data were incomplete and the fully adjusted model had a reduced sample size. Nevertheless, these findings support the concept that functional limitation in ATTR-CM is multifactorial and may not be adequately captured by left ventricular ejection fraction or atrial size alone.

The clinical implications of these findings are relevant. In ATTR-CM, assessment of functional capacity is increasingly important as disease-modifying therapies and improved diagnostic strategies shift attention toward patient-centered outcomes, symptom burden and preservation of physical performance [26]. Our data suggest that atrial fibrillation status may help identify patients with impaired submaximal functional capacity, particularly when evaluated using the 6MWT. This may be relevant for clinical follow-up, rehabilitation referral, rhythm-management discussions and future studies investigating whether targeted management of atrial fibrillation can improve functional outcomes in ATTR-CM. However, the present study was observational and cannot determine whether atrial fibrillation directly causes reduced walking distance or whether it represents a marker of more advanced or heterogeneous disease.

### 4.1 Limitations

First, this was an observational cohort study, and residual confounding cannot be excluded. Second, despite a relatively large ATTR-CM cohort for a functional testing study, subgroup size was limited, particularly for paroxysmal atrial fibrillation. Third, atrial fibrillation classification was based on clinical documentation and rhythm history; therefore, atrial fibrillation burden could not be quantified, except for a small number of patients with implanted cardiac rhythm-devices. Fourth, data on echocardiography and biomarkers were incomplete, reducing sample size in exploratory disease-severity models. Fifth, CPET-derived analyses were secondary and exploratory, and the study was not powered to detect small independent differences across multiple CPET endpoints. Finally, the findings require external validation in larger multicentre ATTR-CM cohorts with systematic rhythm monitoring and longitudinal functional follow-up.

In addition, baseline 6MWD was unavailable in 20/156 patients (12.8%), and the reasons could not be established retrospectively. The higher proportion of NYHA class III among patients with recorded NYHA class and the greater extent of missing NYHA data in patients without baseline 6MWD indicate that selection bias in the complete-case 6MWD analyses cannot be excluded. Gait abnormalities have been described in ATTR-CM [27]. Unrecorded gait or balance difficulties might have limited walking assessments despite the ability to perform seated cycle CPET, which is considered a safer exercise-testing modality than the treadmill in patients with such difficulties [28]. However, this is an unconfirmed hypothesis; the contribution of such factors could not be verified from the available records. The 6MWD findings may therefore not fully represent patients without baseline walking assessments.

## 5 Conclusion

In conclusion, atrial fibrillation status was associated with functional capacity in ATTR-CM, with the most robust association observed for 6MWD. While CPET-derived differences were attenuated after adjustment for clinical parameters, 6MWD remained independently associated with atrial fibrillation status and was directionally consistent across sensitivity analyses. These findings suggest that submaximal functional assessment may capture clinically relevant performance differences across atrial fibrillation status groups in ATTR-CM and support further investigation of rhythm-related functional impairment in this population.

## CRediT authorship contribution statement

**Antonio Kösters:** Writing – original draft, Investigation, Formal analysis, Conceptualization. **Nikita Ermolaev:** Investigation. **Mahshid Eslami:** Data curation. **Lisa Seilinger:** Data curation. **Nima Rassoulpour:** Data curation. **Mariusz Grzeda:** Formal analysis. **Roza Badr Eslam:** Supervision. **Christoph Schukro:** Writing – review & editing, Supervision.

## Funding

This work was supported by institutional research grants from Bayer and AstraZeneca. The funders had no role in the study design, data collection, data analysis, interpretation of results, manuscript preparation, or the decision to submit the manuscript for publication.

## Declaration of competing interest

The authors declare that they have no known competing financial interests or personal relationships that could have appeared to influence the work reported in this paper.

## Appendix A Supplementary data

Supplementary material Table S1: Exploratory regression models examining associations between atrial fibrillation status and functional and biomarker outcomes, with standardized regression coefficients. Table S2: Descriptive comparison of clinical characteristics between patients with and without available baseline six-minute walk distance measurements.
