# Supplementary material for: Atrial fibrillation status and functional capacity in transthyretin cardiac amyloid cardiomyopathy
Source: Int J Cardiol Heart Vasc. 2026 Nov 4;67:102037. doi: 10.1016/j.ijcha.2026.102037 (PMC13760370; doi:10.1016/j.ijcha.2026.102037)
Supplement: Supplementary file 1 [file mmc1.docx]

Supplementary Tables

Atrial Fibrillation Status and Functional Capacity in Transthyretin Cardiac Amyloid Cardiomyopathy

Kösters et al. | IJC Heart & Vasculature | IJCHA-D-26-00720

**Supplementary Table S1. Exploratory secondary endpoint models with standardized coefficients.**

| Outcome | N | Overall AF P | Paroxysmal AF std. β (95% CI) | P value | Permanent AF std. β (95% CI) | P value |
| --- | --- | --- | --- | --- | --- | --- |
| 6MWD | 133 | 0.031 | -0.54 (-0.95 to -0.13) | 0.010 | -0.08 (-0.40 to 0.24) | 0.628 |
| Peak VO₂, mL/kg/min | 148 | 0.154 | -0.21 (-0.61 to 0.19) | 0.298 | -0.31 (-0.63 to 0.01) | 0.058 |
| Peak VO₂, % predicted | 148 | 0.409 | -0.15 (-0.59 to 0.28) | 0.490 | -0.24 (-0.59 to 0.12) | 0.189 |
| Peak workload | 148 | 0.072 | -0.22 (-0.58 to 0.14) | 0.227 | -0.33 (-0.62 to -0.04) | 0.024 |
| Peak workload, % predicted | 148 | 0.572 | -0.12 (-0.51 to 0.28) | 0.566 | -0.17 (-0.49 to 0.15) | 0.302 |
| VE/VCO₂ slope | 146 | 0.179 | 0.18 (-0.26 to 0.62) | 0.420 | 0.34 (-0.02 to 0.70) | 0.065 |
| log NT-proBNP | 98 | 0.007 | 0.40 (-0.12 to 0.93) | 0.132 | 0.66 (0.25 to 1.06) | 0.002 |

Standardized coefficients allow comparison of effect sizes across outcomes. Models were adjusted for age, sex, body mass index and NYHA functional class. Values are standardized β coefficients with 95% CIs. Detailed exploratory results should be interpreted as hypothesis-generating. AF, atrial fibrillation; BMI, body mass index; CI, confidence interval; NT-proBNP, N-terminal pro-B-type natriuretic peptide; NYHA, New York Heart Association; VE/VCO₂, ventilatory equivalent for carbon dioxide; VO₂, oxygen uptake; β, standardized regression coefficient; 6MWD, six-minute walk distance.

**Supplementary Table S2. Characteristics according to availability of baseline six-minute walk distance.**

| **Characteristic** | **Baseline 6MWD available (n=136)** | **Baseline 6MWD unavailable (n=20)** |
| --- | --- | --- |
| Age at CPET, years | 77.6 ± 10.2 (n=136) | 75.5 ± 10.5 (n=20) |
| Male sex | 123/136 (90.4%) | 16/20 (80.0%) |
| Body mass index, kg/m² | 25.5 ± 3.6 (n=136) | 25.8 ± 4.6 (n=20) |
| Wild-type ATTR-CM | 126/136 (92.6%) | 17/20 (85.0%) |
| No atrial fibrillation | 71/136 (52.2%) | 11/20 (55.0%) |
| Paroxysmal atrial fibrillation | 21/136 (15.4%) | 3/20 (15.0%) |
| Permanent atrial fibrillation | 44/136 (32.4%) | 6/20 (30.0%) |
| NYHA class I | 37/133 (27.8%) | 3/15 (20.0%) |
| NYHA class II | 73/133 (54.9%) | 5/15 (33.3%) |
| NYHA class III | 23/133 (17.3%) | 7/15 (46.7%) |
| NYHA class unavailable | 3/136 (2.2%) | 5/20 (25.0%) |
| NT-proBNP, ng/L | 1829 [833, 3222] (n=89) | 2160 [1042, 3202] (n=12) |
| eGFR, mL/min/1.73 m² | 78.6 ± 13.9 (n=94) | 82.3 ± 11.1 (n=16) |
| LVEF, % | 51.9 ± 11.2 (n=75) | 47.7 ± 14.1 (n=11) |
| Peak VO₂, mL/kg/min | 15.4 ± 4.5 (n=136) | 14.4 ± 4.6 (n=20) |
| Peak workload, W | 82.1 ± 31.2 (n=136) | 72.2 ± 38.5 (n=20) |
| Peak respiratory exchange ratio | 1.11 ± 0.10 (n=136) | 1.10 ± 0.10 (n=20) |
| VE/VCO₂ slope | 38.3 ± 8.1 (n=134) | 39.3 ± 9.7 (n=20) |

Values are mean ± SD (available n), median [IQR] (available n) for NT-proBNP, or n/N (%). Age was calculated at the CPET assessment. For NYHA classes I–III, percentages use patients with recorded NYHA class as the denominator; the missing-NYHA row uses the full group denominator. This comparison is descriptive; no hypothesis tests were performed. AF, atrial fibrillation; ATTR-CM, transthyretin cardiac amyloid cardiomyopathy; CPET, cardiopulmonary exercise testing; eGFR, estimated glomerular filtration rate; IQR, interquartile range; LVEF, left ventricular ejection fraction; NT-proBNP, N-terminal pro-B-type natriuretic peptide; NYHA, New York Heart Association; SD, standard deviation; VE/VCO₂, ventilatory equivalent for carbon dioxide; VO₂, oxygen uptake; 6MWD, six-minute walk distance.
